# Expression and functional analysis of *GnRH* at the onset of puberty in sheep

**DOI:** 10.5194/aab-65-249-2022

**Published:** 2022-07-20

**Authors:** Jihu Zhang, Chenguang Wang, Xiaojun Li, Yongjie Zhang, Feng Xing

**Affiliations:** 1 College of Animal Science and Technology, Tarim University, Alar, Xinjiang 843300, China; 2 Key laboratory of Tarim, Animal Husbandry Science and Technology, Xinjiang Production & Construction Corps, Alar, Xinjiang 843300, China

## Abstract

Gonadotropin-releasing hormone (GnRH) is a key factor at the onset of puberty. This decapeptide has been found in mammalian ovaries, but its regulatory mechanism in the ovary of sheep at the onset of puberty is not clear. This study investigated the coding sequence (CDS) of the GnRH gene in the ovary of Duolang sheep and the expression of *GnRH* mRNA in different tissues at the onset of puberty, and analyzed the effect of GnRH on ovarian granulosa cells (GCs) of Duolang sheep. The results showed that the *GnRH* CDS of sheep was cloned, the full length of the *GnRH* CDS in sheep ovary was 279 bp, and the nucleotide sequence was completely homologous to that in the hypothalamus. The expression of *GnRH* mRNA was highest in the hypothalamus and ovary. The expression of related hormones and receptors in GCs of Duolang sheep treated with different concentrations of GnRH for 24 h was affected. GnRH significantly inhibited LH synthesis and *LHR* expression in GCs. Low concentration (100 ng mL ${}^{-1}$ ) had the most obvious therapeutic effect on follicle-stimulating hormone (FSH) and *FSHR*. Higher concentration (250 ng mL ${}^{-1}$ ) significantly promoted estradiol and *ER* β mRNA. These findings provide strong evidence that ovarian GnRH is an important regulatory factor at the onset of puberty in sheep.

## 1 Introduction

In animals puberty is the period of first estrus and ovulation (Spaziani et al., 2020); the animal eventually acquires the ability to reproduce (Witchel et al., 2021). Puberty is considered to be an important marker of the activation of the hypothalamic–pituitary–ovarian axis (HPO) (Smith et al., 2010). Hypothalamic gonadotropin-releasing hormone (GnRH) neurons are the key regulators of puberty and fertility (McIlwraith et al., 2020). At the beginning of puberty, GnRH can activate the established gonad axis (Voliotis et al., 2019). GnRH is secreted from the hypothalamus to the anterior pituitary gland in a pulsating manner during puberty, promoting the synthesis and secretion of follicle-stimulating hormone (FSH) and luteinizing hormone (LH) (Mijiddorj et al., 2017). FSH and LH reach the ovary via blood circulation to promote ovarian development and induce estrogen synthesis in the ovary (Dubois et al., 2002).

At present, the *GnRH* gene and molecular structure of many species have been elucidated (Gao et al., 2020; Nie et al., 2021), and this decapeptide (pGlu–His–Trp–Ser–Tyr–Gly–Leu–Arg–Pro–Gly–NH2) is a key regulator of reproductive functions (Wu et al., 2019; Flanagan et al., 2017). GnRH in mammals is exactly the same and is formed by removing signal peptide from N-terminal and GnRH-associated peptide (GAP) from C-terminal through enzymatic hydrolysis after synthesis of precursor molecules (Limonta et al., 2018). If the sixth position of the GnRH molecule is replaced with Asp, a agonist with higher activity than natural GnRH can be produced. Conversely, an antagonist that reduces activity is produced by substituting Leu acids with Asp except for the sixth position. Both agonists and antagonists can be used to control reproductive activity in animals. The use of exogenous GnRH will affect not only the regulation of hypothalamic–pituitary processes but also other physiological processes related to reproduction (Zanetti et al., 2020). Khalaf et al. (2010) found that the ovarian granulosa cells (GCs) with the treatment of GnRH antagonist could significantly reduce the content of estradiol (E2). GnRH agonists can affect the expression of gonadotropin receptors in the ovary in prepubertal female cats (Mehl et al., 2017). GnRH and its receptors are expressed in many non-hypothalamic reproductive tissues, such as the ovary, uterus, and fallopian tube (La et al., 2010; Peng et al., 2015). As an important reproductive organ, the ovary plays an important role at the onset of puberty. GnRH can affect ovarian function and participate in related reproductive activities, such as regulating follicular development, steroid synthesis, and ovarian epithelial cell proliferation (Fallah et al., 2020). The GCs are the somatic cells around oocytes in the follicle (Tu et al., 2020), whose proliferation and differentiation directly affect ovarian functional activities such as follicle growth and development, ovulation, luteum formation, and hormone secretion. However, whether ovarian *GnRH* directly regulates follicular activity is still unknown.

As a representative breed in Xinjiang, China, Duolang sheep (DL) have the advantage of early puberty (Xing et al., 2019). Therefore, this study took Duolang sheep as the research object, cloned the coding sequence (CDS) of the *GnRH* gene of an ovary, detected the expression of *GnRH* mRNA in tissues at the onset of puberty, and analyzed the effect of GnRH on GCs in vitro. We also investigated the levels of related hormones (FSH, LH, and E2) and the expression of hormone receptors (*GnRHR*, *LHR*, *FSHR*, *ER* α , and *ER* β ) in GCs treated with different concentrations of GnRH.

## 2 Materials and methods

### 2.1 Animals and tissue collection

The selected sheep were raised in the Tarim University experimental station (40 ${}^{\circ }$ 33 ${}^{\prime }$ 2 ${}^{\prime \prime }$ N, 81 ${}^{\circ }$ 17 ${}^{\prime }$ 4 ${}^{\prime \prime }$ E; altitude 1101 m a.s.l. (above sea level)). Female sheep in prepuberty (145 d age), puberty (within 4–6 h of the first natural estrus), and post-puberty (3 d after the end of estrus) periods were selected. Each period was represented by four sheep, which were of the same strain and reared under the same conditions.

We identified pubertal sheep by detecting changes in estrus and in the vulva. Sheep were deeply anesthetized by intravenous administration of 3 % pentobarbital sodium (Solarbio, Beijing, China) and sacrificed by exsanguination at a healthy physiological stage. After death, the hypothalamus, pituitary gland, ovary, uterus, fallopian tube, heart, liver, spleen, lung, and kidney were removed. After being cut into pieces by sterile surgical scissors, they were placed in cryo-preservation tubes and quickly preserved in liquid nitrogen.

### 2.2 Culture of granulosa cells from the ovary

Fresh and healthy DL ovaries in puberty were rinsed with 75 % alcohol and then rinsed with phosphate-buffered saline (PBS) three times to completely remove residual alcohol. In Dulbecco's modified eagle medium (DMEM) (Invitrogen, Carlsbad, USA) culture medium, a sterile medical syringe needle was inserted into the follicular cavity of the ovary to absorb the follicular fluid. The follicular fluid and culture medium were mixed and injected into a 15 mL sterile centrifuge tube. The supernatant was discarded after centrifugation at 1000 rpm for 5 min. Following this, 3 mL of DMEM was added, and the mixture was then evenly mixed by light stirring and centrifuged at 1000 rpm for 5 min, after which the supernatant was discarded. The newly prepared and complete DMEM culture medium (10 % fetal bovine serum (FBS) + 1 % double antibody) (Invitrogen, Carlsbad, USA) was added, mixed, and transferred to petri dishes. Samples were placed in an incubator and cultured at 37 ${}^{\circ }$ and 5 % CO ${}_{2}$ for 24 h. Next the samples were treated by different concentrations (0, 50, 100, 200, 250 and 500 ng mL ${}^{-1}$ ) of GnRH (gonadorelin, Sansheng, Ningbo, China). After 24 h, the RNA of GCs was extracted, and the supernatant was then used for enzyme-linked immunosorbent assay (ELISA) (LH, E2, and FSH) (Jining, Shanghai, China).

### 2.3 Total RNA isolation and cDNA synthesis

Trizol (Invitrogen, Carlsbad, USA) was used to extract RNA, and the concentration of extracted RNA was detected using a nucleic acid protein detector (Denovix, Wilmington, USA). The RNA concentration was determined by measuring absorbance at 260 nm. The purity of all RNA was assessed as the ratio of absorbance at 260 and 280 nm (A260 and A280). The cDNA was synthesized using a PrimeScript™ RT reagent kit (TaKaRa, Beijing, China). According to the manufacturer's instructions, the amplification was carried out in a reaction system of 20 µ L. The reaction mixture was incubated for 50 min at 42 ${}^{\circ }$ C, followed by 5 min at 95 ${}^{\circ }$ C to deactivate the reverse transcriptase.

### 2.4 Cloning of sheep GnRH gene

The polymerase chain reaction (PCR) was performed in a 25 µ L total volume, containing 1 µ L of ovarian cDNA, 0.5 µ L of each primer (GnRH-1F/R, 10 µ M, Table 1), and 12.5 µ L of PCR mix (TaKaRa, Beijing, China). Reverse transcription PCR (RT-PCR) with primers (Table 1) was treated as follows: it was treated at 95 ${}^{\circ }$ for 5 min, followed by 35 cycles of 95 ${}^{\circ }$ for 30 s, and annealed for 30 s at 72 ${}^{\circ }$ for 1 min, followed by one cycle at 72 ${}^{\circ }$ for 5 min. The RT-PCR product was ligated with PMD-19T (TaKaRa, Dalian, China). The well-reacted ligands were mixed with DH5 α receptor cells (Trans, Beijing, China). The recombinants were selected by a blue-white spot (Solarbio, Beijing, China) reagent and amplified by PCR. The qualified bacterial solution was sent for sequencing.

**Table 1:** Primers used for cloning the CDS of the GnRH gene and qPCR.

Primer	Primer	Product	Annealing
	sequence	size	temperature
	(5 ${}^{\prime }$ –3 ${}^{\prime }$ )	(bp)	( ${}^{\circ }$ C)
*GnRH*-1	F: CCAGTCTGGGTCTATTTTTATCACT	322	54
	R: GTGGATTTATATTTTCCTCTGCCCAG		
*GnRH*-2	F: CCTGCTGACTTTCTGTGTGG	148	60
	R: TCTACTGGCTGATCGACCTC		
*GnRHR*	F: AACAGCAGCATCCTACTAACACCG	158	58
	R: CTCTTCTCTTTCCTTTGAGCCCA		
*LHR*	F: TGCTTACCCAAGACACTC	101	62
	R: ATCAGCCAAATCAGGAC		
*FSHR*	F: CTTGCCAGCTGTTCACAAGA	190	60
	R: CTCATCGAGTTGGGTTCCAT		
*ER* α	F: CAGGGAAGCTCCTATTTGCTC	182	60
	R: GTACACCCCAGAATTAAGCAAG		
*ER* β	F: TGCTGCTGGAGATGCTGAATG	112	60
	R: GGTTTCTGGGAGCCCTCTTTG		
β -*actin*	F: CACGGTGCCCATCTACGA	158	60
	R: TTTAGCAGGCACTGTAGTTCC		

*GnRH*: XM_015093089.1. *GnRHR*: NM_ 001009397.1. *LHR*: NM_001278566.2. *FSHR*: NM_001009289.1. *ER* α : XM_042253635.1. *ER* β : NM_001009737.1. *ACTB* ( β *-actin*): NM_001009784.3. Primers are based on the information from NCBI (https://www.ncbi.nlm.nih.gov, last access: 10 September 2021).

### 2.5 Reverse transcription polymerase chain reaction

The RT-PCR was performed to examine the *GnRH* expression profile in the hypothalamus, pituitary gland, ovary, fallopian tube, uterus, liver, heart, kidney, lung, and spleen. PCR was performed in a 25 µ L total volume solution containing 1 µ L of cDNA, 0.5 µ L of each primer (10 µ M, Table 1), and 12.5 µ L of PCR mix (TaKaRa, China). The conditions were the same as cloning. *ACTB* ( β -*actin*) was used as internal reference, and PCR products were run separately on 1.5 % agarose gels.

**Figure 1:**
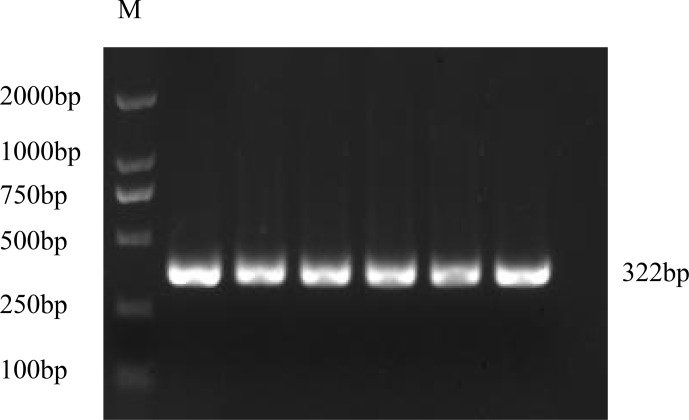
Cloning of the CDS of the *GnRH* gene. M represents DL2000 Marker.

### 2.6 Quantitative real-time PCR

Using cDNA as a template, the expression of *GnRH* mRNA in hypothalamus, ovary, uterus, and fallopian tube tissue and *GnRHR*, *ER* α, *ER* β , *FSHR*, and *LHR* in GCs were detected using PerfectStart Green qPCR SuperMix (Trans, Beijing, China). Quantitative real-time PCR (qPCR) was performed using 7.5 µ L of 2 × Transtant qPCR mix, equimolar amounts of forward and reverse primers (0.5 µ L, 10 µ M, Table 1), and 1 µ L of diluted cDNA ( 1:2 in RNase-free water) in a final volume of 15 mL. The qPCR was performed under the following conditions: it was treated at 95 ${}^{\circ }$ for 10 min, followed by 40 cycles at 95 ${}^{\circ }$ for 20 s and 60 ${}^{\circ }$ for 30 s and then treated at 95 ${}^{\circ }$ for 15 s, 60 ${}^{\circ }$ for 30 s, and 95 ${}^{\circ }$ for 15 s. Each sample was run three times.

### 2.7 Statistical analysis

The standard curve was made after the supernatant reproductive hormone concentration was obtained. Cycle threshold (CT) values were calculated using the 2 ${}^{-\Delta \Delta ct}$ method (Livak et al., 2001). SPSS 24.0 was used to analyze whether the difference was significant. The difference was considered significant at p<0.05 .

## 3 Results

### 3.1 Cloning of CDS of the *GnRH* gene

The PCR product of the *GnRH* CDS was detected by 1.5 % agarose gel electrophoresis (Fig. 1). The target fragments of *GnRH* were consistent with the prediction.

### 3.2 Similarity comparison and phylogenetic tree construction

**Figure 2:**
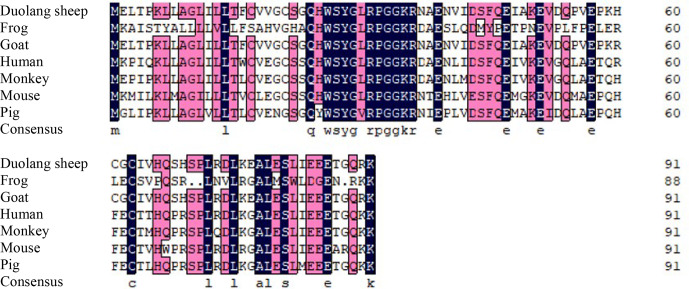
Alignment of the amino acid sequences of Duolang sheep GnRH with those of a monkey (NM_001195436.2), pig (NM_001172956.1), goat (XM_013966090.2), frog (XM_ 041587005.1), mouse (NM_008145.3), and human (NM_000825.3).

**Figure 3:**
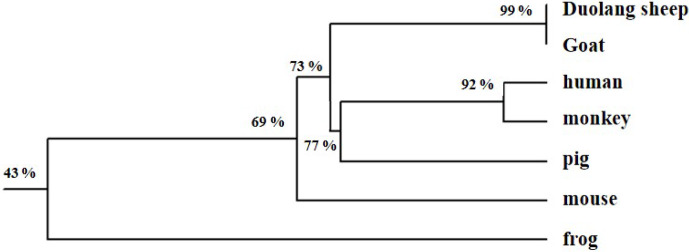
Phylogenetic tree of the *GnRH* gene amino acid sequence.

DNAMAN 6.0 was used to deduce that the *GnRH* CDS of Duolang sheep encoded a 92 amino acid protein. The molecular formula was C ${}_{446}$ H ${}_{722}$ N ${}_{124}$ O ${}_{136}$ S ${}_{6}$ , and the total molecular weight was 10.19 kDa. ExPASy predicted that the total average hydrophilicity of the protein was - 0.218, indicating that the protein was hydrophilic. The isoelectric point was 5.55. The amino acid sequence of *GnRH* of Duolang sheep was compared with those of a monkey, pig, goat, frog, mouse, and human (Fig. 2). Phylogenetic tree analysis showed that the *GnRH* of Duolang sheep had the closest relationship with the goat (99 %) and the farthest relationship with the frog (43 %) (Fig. 3).

**Figure 4:**
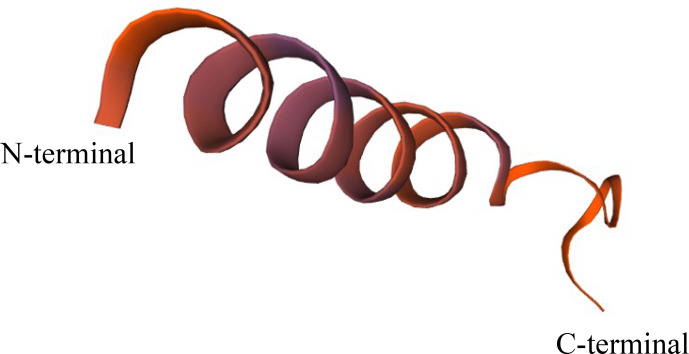
Prediction of the tertiary structure of GnRH protein.

The secondary structure of the *GnRH* protein was analyzed by SOPMA software. The secondary structure was mainly composed of α -helix and random coil structures (45.65 % and 39.13 %, respectively), and the rest was made up of extended chain and β -rotation structures (11.96 % and 3.26 %, respectively). A Swiss model was used to predict the tertiary structure of the protein (Fig. 4). This model was established based on template 6F0K.1.g, and the seq similarity was 0.35.

### 3.3 Expression profile of the *GnRH* gene in sheep tissues

**Figure 5:**
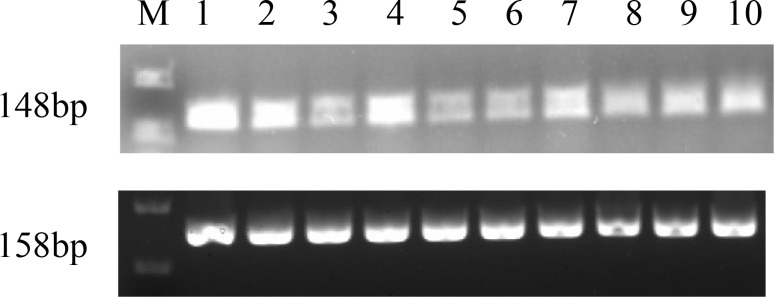
Expression profile of the *GnRH* gene in sheep tissue.

The expression profiles of *GnRH* in different tissues at the pubertal stage were assessed (Fig. 5). The results showed that the expression of *GnRH* was relatively high in the hypothalamus, ovary, and fallopian tube, and weak expression was found in pituitary, uterus, heart, liver, spleen, lung, and kidney tissue.

**Figure 6:**
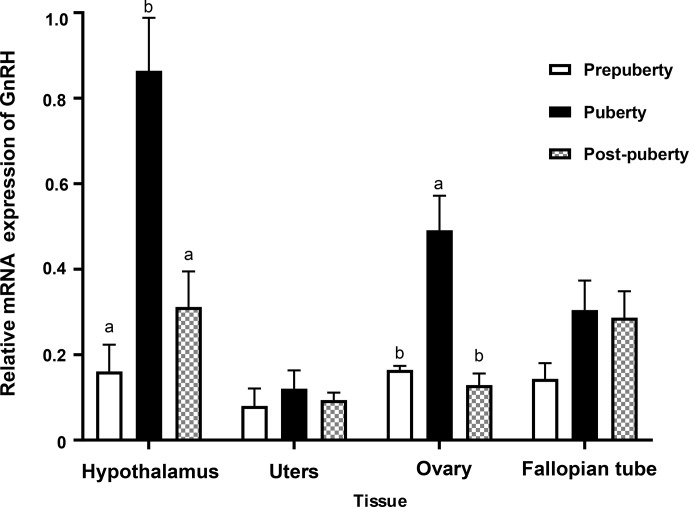
Relative mRNA expression of *GnRH* at different pubertal stages.

**Figure 7:**
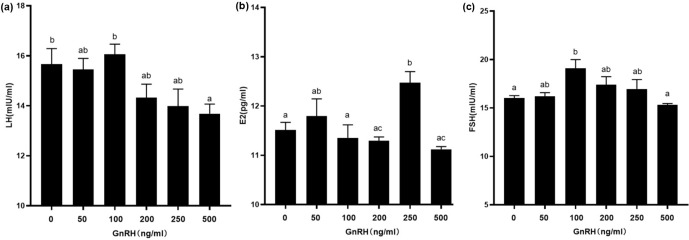
Contents of LH, E2, and FSH in GCs at different GnRH concentrations. Bars with different lowercase letters are significantly different at p<0.05 , n=3 .

**Figure 8:**
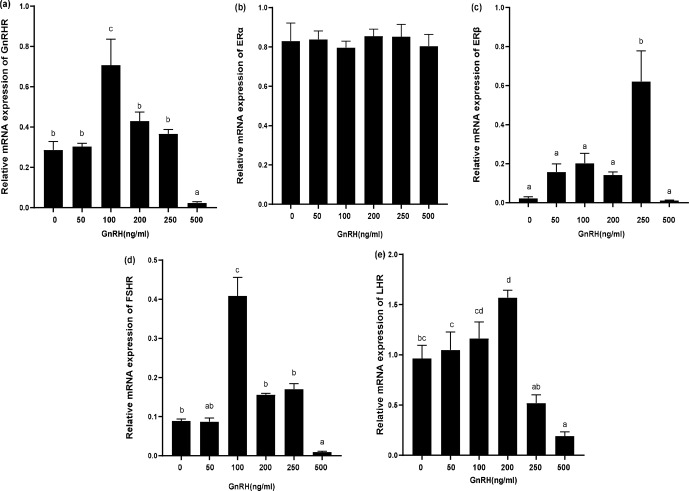
Relative mRNA expression of *GnRHR*, *LHR*, *FSHR*, *ER* α , and *ER* β in GCs treated with different GnRH concentrations. Bars with different lowercase letters are significantly different at p<0.05 , n=3 .

M-DL 2000 markers are as follows: (1) hypothalamus, (2) ovary, (3) pituitary, (4) fallopian tube, (5) uterus, (6) heart, (7) liver, (8) spleen, (9) lung, and (10) kidney.

### 3.4 The expression of *GnRH* in tissues at different pubertal stages

In order to study the role of *GnRH* gene in the reproductive system of Duolang sheep at the onset of puberty, the expression of *GnRH* mRNA in the hypothalamus, uterus, ovary, and fallopian tube at different pubertal stages was detected by qPCR (Fig. 6). The expression of the hypothalamus was the highest, followed by the ovary and fallopian tube. The expression of *GnRH* mRNA in the hypothalamus was significantly higher from prepuberty to puberty, and then there was a rapid decline in post-puberty ( p<0.05 ). The expression of *GnRH* mRNA in ovary was second only to the hypothalamus, which was significantly increased in prepuberty ( p<0.05 ) and significantly decreased in post-puberty ( p<0.05 ). *GnRH* mRNA was expressed in uterus and fallopian tube, but it was stable in three stages ( p>0.05 ).

Bars with different lowercase letters of the same tissue are significantly different at p<0.05 , n=4 .

### 3.5 Effects of exogenous GnRH on LH, E2, and FSH in GCs

In order to study the effects of GnRH on related hormones in ovarian GCs, we treated GCs in puberty of Duolang sheep with different concentrations of GnRH for 24 h (Fig. 7). ELISA showed that high GnRH concentration (500 ng mL ${}^{-1}$ ) significantly inhibited LH ( p<0.05 ). GnRH significantly promoted E2 at 250 ng mL ${}^{-1}$ ( p<0.05 ) and promoted FSH at 100 ng mL ${}^{-1}$ ( p<0.05 ), but it had no obvious inhibitory effect at high concentration ( p>0.05 ).

### 3.6 Effects of GnRH on expression of *GnRHR*, *LHR*, *FSHR*, *ER* α , and *ER* β in GCs

The expression of relevant hormone receptors was detected in GCs with different concentrations of GnRH by qPCR (Fig. 8). The results showed that *ER* α was not affected by GnRH (Fig. 8b). GnRH significantly inhibited *GnRHR*, *FSHR*, *LHR*, and *ER* β at high concentrations (500 ng mL ${}^{-1}$ ). Relative mRNA expression of *GnRHR* was the highest at 100 ng mL ${}^{-1}$ and gradually decreased with the increase of GnRH concentration (Fig. 8a). *ER* β was most significantly promoted at 250 ng mL ${}^{-1}$ (Fig. 8c) and *FSHR* was most significantly promoted at 100 ng mL ${}^{-1}$ (Fig. 8d). GnRH had a significant inhibitory effect on *LHR* at 500 ng mL ${}^{-1}$ (Fig. 8e).

## 4 Discussion

The CDS of *GnRH* gene in the ovary of Duolang sheep was cloned, and the similarity comparison showed that it had the highest similarity with goat and the lowest similarity with frog. The CDS of the *GnRH* gene was relatively conserved and was consistent with the characteristics of biological evolution. *GnRH* gene of sheep consists of three exons and two introns, and the CDS was 279 bp in length, encoding 92 amino acids. Bioinformatics analysis showed that *GnRH* protein was acidic with an isoelectric point of 5.55. The instability index was 51.75, belonging to unstable protein. The total average hydrophilicity was - 0.218, which was a hydrophilic protein, consistent with its transcription pattern. The main secondary structure of the protein was α -helix and random coil, which can ensure the protein generates a more stable tertiary structure, provide enzyme activity sites and other protein-specific functional sites, and generate a specific spatial conformation and tertiary structure.

GnRH was most expressed in the hypothalamus, indicating that it played a key role at the onset of puberty. The combination of *GnRH* secreted by hypothalamus and *GnRHR* on pituitary tissue activated the Gq and G11 subfamily of G-protein (Ying et al., 2020) and ultimately promotes the synthesis and release of gonadotropin, thus initiating the puberty. Due to the rapid decomposition of GnRH in the body and its massive dilution in blood circulation, the role of GnRH in the hypothalamus is limited to the pituitary tissue (Litichever et al., 2009). Therefore, the reproductive function of GnRH outside the hypothalamus has attracted special attention in recent years. We studied *GnRH* mRNA in 10 types of tissue from Duolang sheep, indicating that it can function according to the type of target tissues and physiological conditions. The relative mRNA expression of *GnRH* in the ovary of Duolang sheep was second only to that in the hypothalamus, and the expression trend was consistent with that in the hypothalamus, suggesting ovarian *GnRH* also played an important role at the onset of puberty. *GnRH* mRNA in the ovaries of ducks was significantly increased from prepuberty to puberty (Ni et al., 2007). In situ hybridization revealed the localization of *GnRH* mRNA in primary, secondary, and tertiary GCs (Clayton et al., 1992; Whitelaw et al., 1995). The rapid decrease of *GnRH* mRNA in ovary from puberty to post-puberty indicated that *GnRH* was related to luteal formation after ovulation. Choi et al. (2006) also found that *GnRH* and *GnRHR* were strongly expressed in human corpus luteum granulosa cells. These studies suggest that *GnRH* is an important autocrine and paracrine regulator in the ovary.

It has been confirmed that GnRH and its receptor exist in animal ovaries and play important physiological roles in animal reproductive cycle (Schirman-Hildesheim et al., 2005; Singh et al., 2008). In the ovary, *GnRH* needs to bind to *GnRHR* to work. A common feature of *GnRHR* in mammals is the absence of the carboxy terminal tail present in all other receptors in the G-protein-coupled receptors family (Blomenrohr et al., 1999; Vrecl et al., 2000), resulting in slow receptor internalization and rapid desensitization (Metallinou et al., 2007). The expression of *GnRHR* was the highest when GnRH was 100 ng mL ${}^{-1}$ . With the increase in concentration, the expression of *GnRHR* decreased gradually, indicating that high concentrations of GnRH can inhibit the expression of its receptor and that desensitization may also exist. GnRH regulates the synthesis of steroids, such as estrogen, and also affects ovarian gonadotropin production. In mouse ovaries, low doses of GnRH promoted ovarian steroid production and follicular development and high doses of GnRH inhibited follicular development and ovulation (Singh et al., 2010). It is also associated with the transformation of some genes in follicular maturation and ovulation, such as tissue fibrinogen. GCs with the treatment of GnRH significantly increased the levels of FSH and E2, indicating that GnRH can promote the expression of ovarian FSH and E2. *GnRH* mRNA was high in the ovary in prepuberty, possibly to stimulate the expression of FSH and E2 to promote follicular development. It was obvious that the GnRH had no significant effect on *ER* α expression, while *ER* β changed with the concentration change. Follicles of mice lacking *ER* α still matured and were still able to ovulate (Byers et al., 1997; Couse et al., 1999). Surprisingly, *ER* β -deficient mice failed to develop follicles and ovulate (Jayes et al., 2014). These studies suggested that the main receptor of E2 in follicles was *ER* β . GCs are the main locus of *ER* β regulation gene expression, which expresses *ER* β and is involved in the production of steroids and gonadotropins, and *ER* β is essential for follicular development and ovulation (Lee et al., 2021). However, it is not known whether the increase in *ER* β was caused directly by the exogenous GnRH or the E2 caused by GnRH. E2 and *ER* β production were the highest in GCs at high concentrations (250 ng mL ${}^{-1}$ ) of GnRH. GnRH can promote the formation of *ER* β and E2 and promote follicular development in prepuberty. GnRH increased significantly in puberty and increased E2 and *ER* β expression, which may be related to ovulation. FSH is essential for the ovary to perform its reproductive function. Li et al. (2019) found that *FSHR* was expressed in the follicular stage and luteal stage in two different breeds of sheep in China. GCs have receptor sites for many other hormonal and non-hormonal factors through which FSH can exert stimulatory or inhibitory effects. FSH can activate camp-dependent physiological processes, promote the activity of steroid hormone-producing enzymes in ovarian granulosa cells, and increase E2 in blood. FSH and E2 can jointly promote the proliferation of GCs, the enlargement of follicular cavity, and the development of follicles. The inhibition of LH and *LHR* was obvious at high GnRH concentration (500 ng mL ${}^{-1}$ ). GnRH agonist can significantly inhibit LH expression in rat ovarian GCs (Litichever et al., 2009). GnRH inhibited ovarian *LHR* expression in cats in puberty (Mehl et al., 2017). These findings suggest that ovarian GnRH plays a role by down-regulating LH and *LHR*. The content of *GnRH* mRNA in the ovary was lower from puberty to post-puberty, which may be related to the formation of luteum.

## 5 Conclusions

In general, *GnRH* CDS of Duolang sheep had the highest similarity with *goat*. The expression of *GnRH* in the hypothalamus and ovary was the highest in puberty. In ovarian GCs, GnRH significantly inhibited LH and *LHR*. Moderate GnRH can up-regulate FSH, E2, *FSHR*, and *ER* β . These results suggested that ovarian *GnRH* was involved at the onset of puberty in sheep and may be related to ovarian follicular development and ovulation.

## Data Availability

The original data are available upon request to the corresponding authors.
